# The SGLT2 Inhibitor Canagliflozin Promotes β‐Cell Regeneration and Restores and Stabilises β‐Cell Identity in a Polygenic Model of Severe Early‐Onset Type 2 Diabetes

**DOI:** 10.1111/jcmm.71041

**Published:** 2026-03-11

**Authors:** Iuliana Popescu, Robert C. Bunn, Phil Ray, Kathryn M. Thrailkill, John L. Fowlkes

**Affiliations:** ^1^ Barnstable Brown Diabetes Center College of Medicine, University of Kentucky Lexington Kentucky USA; ^2^ Department of Pediatric Endocrinology College of Medicine, University of Kentucky Lexington Kentucky USA; ^3^ Barnstable Brown Chair in Pediatric Diabetes Research College of Medicine Lexington Kentucky USA

**Keywords:** β‐cell, canagliflozin, paediatric diabetes, pancreatic islet, regeneration, SGLT2

## Abstract

Childhood obesity has led to an increase in type 2 diabetes (T2D) among youth, with adolescent‐onset T2D showing a rapid decline in β‐cell function compared to adult‐onset cases. While the disease progression is more aggressive in early life, treatment can lead to recovery or remission more often at younger ages. SGLT2i have proven multiple health benefits when prescribed to adults with T2D but may have a greater potential in improving insulin production and β‐cell mass in youth. In our study, TallyHO mice, which develop severe early‐onset T2D, were treated with canagliflozin (cana) while on a 10‐week diet. Results showed a significant reduction in blood glucose levels and improved β‐cell function, indicated by higher C‐peptide, islet insulin content, and HOMA‐B index compared to untreated mice. Cana treatment restored the islet area and β to α‐cell ratio, while also decreasing apoptosis. Notably, cana promoted the transient appearance of endocrine bihormonal cells and small clusters of insulin‐positive cells, suggesting a possible transdifferentiation process and β‐cell neogenesis. Furthermore, cana stabilised β‐cell phenotype, restoring the expression of key identity markers while reducing abnormal cell types and the dedifferentiation to precursors and mesenchymal cells. These findings suggest that canagliflozin can promote the regeneration of pancreatic islets and mitigate β‐cell dedifferentiation in the early onset of β‐cell deficiency.

## 1 Introduction

With the rising prevalence of childhood obesity, the incidence of type 2 diabetes (T2D) in the paediatric population continues to grow [1]. Childhood and adolescent obesity negatively impacts early glycemic control and is associated with a faster progression of diabetes‐related complications, leading to higher morbidity and mortality rates in adulthood [2]. Functional studies have shown that youth exhibit more significant insulin resistance and a more rapid decline in β‐cell function compared to adults with similar adiposity and glycemic status [3, 4]; this may be due to defective metabolic flexibility in younger populations.

SGLT2 inhibitors (SGLT2i; ‘gliflozins’) are a relatively new class of oral anti‐hyperglycemic medications used in adults with T2D, either as monotherapy or in combination with other hypoglycemic medications. SGLT2i reduce the reabsorption of glucose from the filtered urine, leading to lower blood glucose levels, sodium depletion, glucosuria, and osmotic diuresis. Clinical trials involving individuals with T2D and studies in animal models treated with gliflozins have highlighted benefits beyond their effects on blood sugar control. These off‐target effects include positive outcomes related to body weight, blood pressure, cardiac remodelling, atherogenesis, glomerular hemodynamics, inflammation, and fibrosis, supporting the role of SGLT2i in cardiovascular and renal protection and in delaying the progression of fatty liver disease [5]. Additionally, pharmacological inhibition of SGLT2, or its deletion/loss‐of‐function, has shown promising outcomes for endogenous insulin production and preservation of β‐cell mass in individuals and animal models with T2D [6], and in a few studies, in type 1 diabetes (T1D) [7, 8]. However, it remains unclear whether the positive effects of SGLT2i on β‐cell function are due to: (1) an off‐target effect on β‐cells (β‐cells do not express the SGLT2 transporter [9]); (2) effects on other islet cell types (e.g., α‐cells [9]) or the exocrine pancreas; (3) indirect effects such as a reduction in peri‐islet glucotoxicity or oxidative/inflammatory stress; (4) alteration of intra‐islet paracrine interactions; or (5) a combination of these mechanisms. Considering the extraordinary heterogeneity and plasticity of pancreatic endocrine cells, the potential for intra‐islet (e.g., α‐cells, δ‐cells) and extra‐islet cells (acinar and ductal) to contribute to β‐cell regeneration in conditions of β‐cell loss [10, 11, 12], and the ability of SGLT2i to slow the progression of β‐cell failure [6], we hypothesized that the SGLT2i canagliflozin (Cana) could play a role in β‐cell endocrine specification and regeneration (particularly evident in early‐onset diabetes) and could also prevent or reverse β‐cell dedifferentiation, a mechanism underlying β‐cell deficits in T2D. To test our hypothesis, we explored the effects of Cana on glucose homeostasis and β‐cell function and mass in vivo and examined islet architecture and endocrine cell fate in male TallyHO/JngJ (TH) mice, a polygenic model of early‐onset T2D that reflects many aspects of T2D in adolescents [13, 14]. Our findings indicate that Cana treatment prevents β‐cell dedifferentiation by stabilising its identity, and enables the regeneration of damaged islets in the early onset of severe β‐cell deficiency. These beneficial effects of Cana lead to significant improvements in β‐cell function and mass, possibly due to the activation of endogenous multiple mechanisms that fine‐tune the homeostatic renewal of pancreatic endocrine cells.

## 2 Material and Methods

### 2.1 Animal Experiments

**FIGURE 1:**
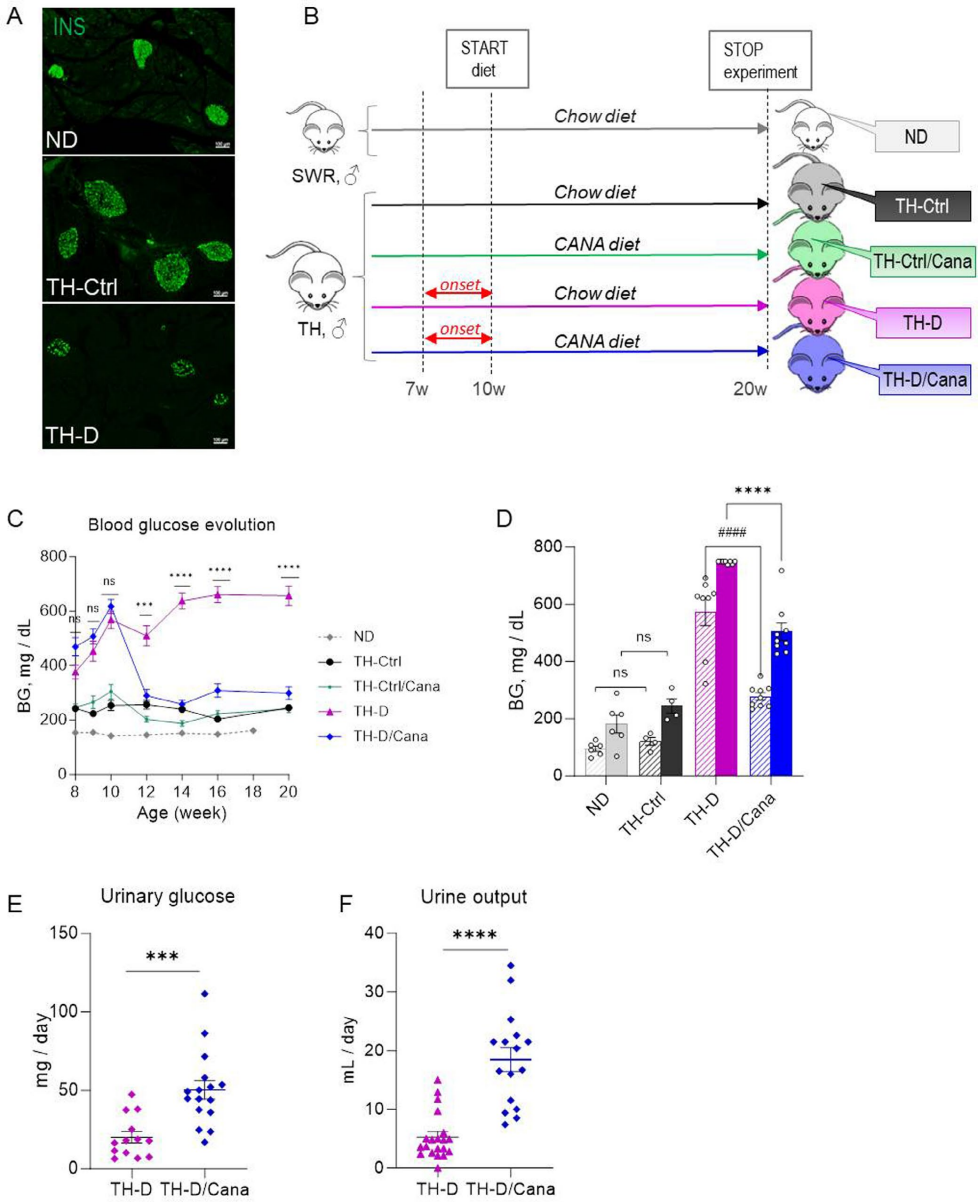
Experimental design and glycemic parameters check. (A) General appraisal of the pancreatic islets in the experimental mouse phenotypes. TH‐Ctrl mice display enlarged islets compared to non‐diabetic SWR mice, while diabetic TH‐D mice display smaller and damaged islets. (B) Male TH mice were randomised at 9 weeks of age based on their individual non‐fasting BG values to receive chow (TH‐Ctrl; TH‐D) or chow‐containing Cana diet (TH‐Ctrl/Cana; TH‐D/Cana) starting with the age of 10 weeks (w) for another 10 weeks. Age‐matched male SWR lean mice fed with a chow diet were the non‐diabetic (ND) control group. (C) Evolution of non‐fasting blood glucose concentrations (BG, mg/dL) measured in plasma, starting with the age of 8 weeks until the end of the study. Data are expressed as mean ± SEM (*n* = 7–17). BG was analysed using a two‐way repeated‐measures ANOVA with time as the within‐subject factor and treatment group as the between‐subject factor. Greenhouse–Geisser correction was applied when sphericity was violated. Post hoc comparisons between groups at each time point were performed using Šídák's multiple‐comparison test (the statistics is shown only for TH‐D and TH‐D/Cana groups; no significant differences between TH‐Ctrl and TH‐Ctrl/Cana mice). (D) BG concentrations were measured in awakening 20‐week‐old mice 16 h post‐fasting (hatched bars) and 4 h post‐feeding (solid bars); TH‐Ctrl/Cana mice were unavailable for this experiment. Data are expressed as mean ± SEM (*n* = 6–9). Two‐way ANOVA with Tukey post hoc test was used to analyse statistical differences between groups. (E) Daily excreted urinary glucose measured at the end of the treatment. (F) Urine volume excreted over 24 h at the end of the treatment. Data are expressed as mean ± SEM (*n* = 14–16). Mann–Whitney test (two‐tailed) was used to analyse statistical differences between groups. *p < 0.05, **p < 0.01, ***p < 0.001, ****p < 0.0001. “ns” means not significant, #### *p* < 0.0001.

**FIGURE 2:**
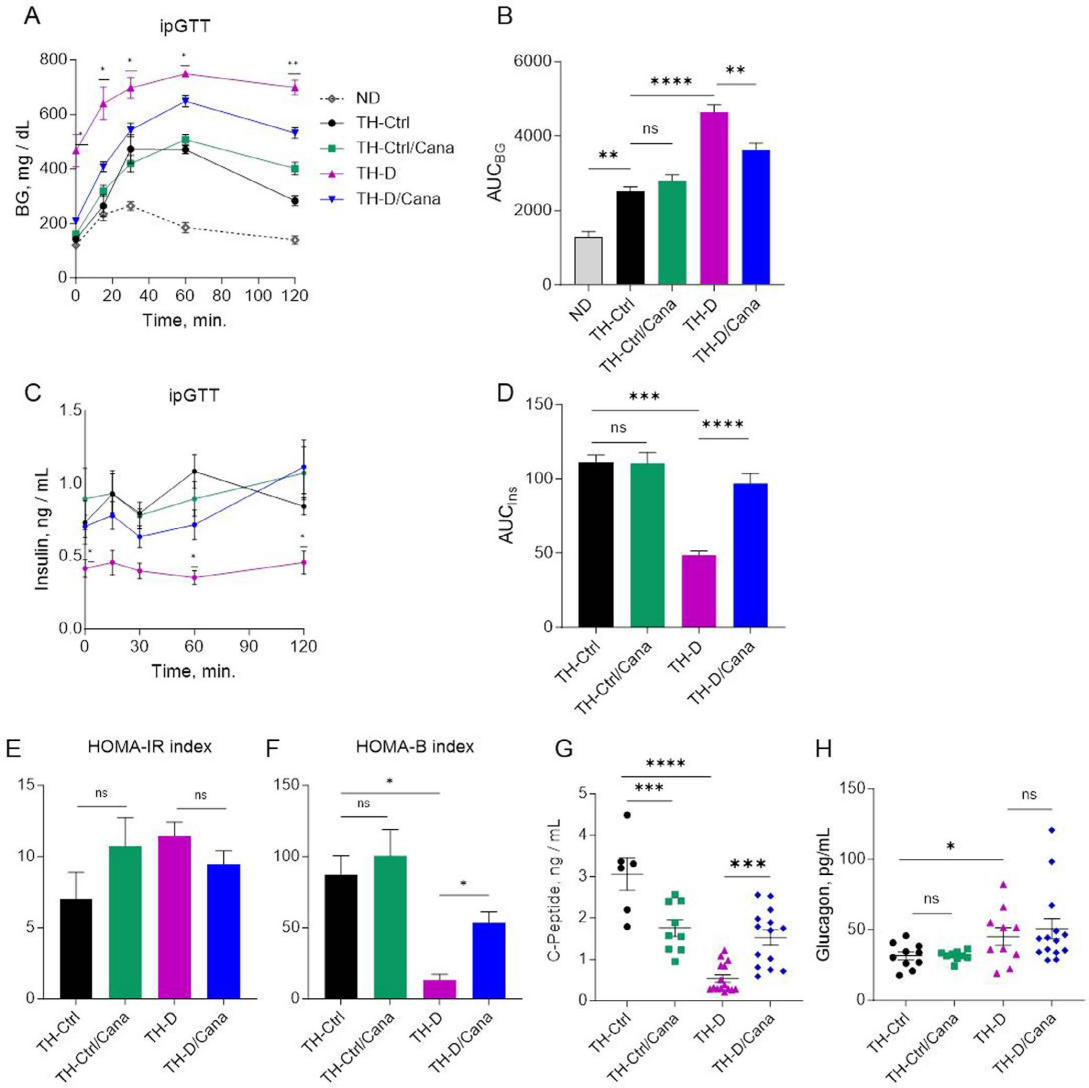
Assessment of in vivo glycemic status upon Cana treatment. (A–D) Plasma glucose and insulin concentrations during ipGTT. (A, C) represent glucose and insulin excursion curves, respectively, after O/N fasting in mice fed a standard chow or Cana diet. Insulin values of the ND mice were not consistently measured in this experiment. Data are expressed as mean ± SEM (*n* = 7–12). A two‐way repeated‐measures ANOVA was applied (the statistics is shown only for TH‐D and TH‐D/Cana groups; no significant differences between TH‐Ctrl and TH‐Ctrl/Cana mice). (B, D) represent the total change in BG or insulin levels over the ipGTT expressed as area under the curve (AUC) for each experimental group. (E, F) HOMA‐IR and HOMA‐B indices were calculated in O/N fasted mice. HOMA‐IR = [FBG × FI (mU/L)]/405; HOMA‐B = [360 × FI(mU/L)]/[FBG – 63]. (G, H) Plasma concentrations of C‐peptide and glucagon were measured at the end of the study by ELISA. Data are expressed as mean ± SEM (*n* = 7–14). One‐way ANOVA followed by Bonferroni's multiple comparison post hoc test was used to analyse statistical differences between groups. *p < 0.05, **p < 0.01, ***p < 0.001, ****p < 0.0001. “ns” means not significant.

We used TallyHO (TH) male mice, a polygenic model of T2D that develops mild obesity and early‐onset diabetes characterised by a rapid decline in β‐cell function and mass and severe hyperglycemia [14] (Figure 1A); female TH mice, which maintain a normal blood glucose range, provide a model of euglycemic mild obesity and do not manifest overt diabetes and β‐cell failure [14]. TH mice were randomised to receive a standard chow or [chow + canagliflozin 100 ppm] diet for 10 weeks (Figure 2B). Animal handling and experimental design are further detailed in the Supporting Information.

### 2.2 Intraperitoneal Glucose Tolerance Test (ipGTT)

For the Glucose Tolerance Test, 16‐h‐fasted mice (overnight, O/N) were given an intraperitoneal (ip) injection of glucose in saline solution (1.5 g/kg body weight); blood glucose was measured with a glucometer at 0, 15, 30, 60, and 120 min after the glucose injection. Blood samples were collected in capillary blood collection tubes with EDTA (Sarstedt Microvette, Fisher Scientific) at the same intervals for the subsequent assessment of plasma insulin levels.

### 2.3 Biochemistry

Insulin, C‐Peptide, and Glucagon hormones were assessed in the blood plasma of mice by ELISA, as previously described [14, 15].

Measurement of urine output and the assay of urinary glucose were performed as previously described [15].

### 2.4 Pancreatic Insulin Content

Pancreas insulin was extracted by the acidic‐ethanol method and assayed for insulin concentration by an ELISA protocol (Crystal Chem, Elk Grove Village, IL). Insulin concentrations were normalised to the protein content measured by the Bradford method.

### 2.5 Apoptosis Detection

TUNEL staining was performed using Millipore ApopTag Peroxidase in situ apoptosis detection kit (S7100, Sigma‐Aldrich) according to the manufacturer's recommendations. Appropriate positive and negative controls were run simultaneously. The quantification of the TUNEL+ area was performed by the threshold method in ImageJ 1.52a. Immunohistochemistry, super‐resolution microscopy and histomorphometry—these methods are described in the Supporting Information.

### 2.6 Statistical Analysis

Data are presented as the mean ± SEM. Mann–Whitney test or One‐way or Two‐way ANOVA (with multiple comparison post hoc tests as indicated in the figure legend) were performed for between‐groups analysis using GraphPad Prism (version 10.1.0 for Windows), GraphPad Software, Boston, Massachusetts, USA (www.graphpad.com). A *p* value < 0.05 was considered statistically significant. *p < 0.05, **p < 0.01, ***p < 0.001, ****p < 0.0001.

## 3 Results

### 3.1 Canagliflozin Induced Glucosuria, Elevated Urine Output, and Curtailed Blood Glucose Levels in Severely TH Diabetic Mice

Almost 70% of TH male mice developed overt diabetes by 8 weeks of age, showing increased non‐fasting blood glucose (BG) levels (377.06 ± 25.6 mg/dL; TH‐D). Non‐converters maintained lower BG levels (243 ± 6.2 mg/dL; TH‐Ctrl) until the study's end but were still higher than control normoglycemic SWR mice (153.75 ± 4.9 mg/dL; ND). Canagliflozin rapidly reduced BG levels in TH‐D mice (−53%) after only 2 weeks of treatment (*p* < 0.001), while untreated mice's BG continued to rise above 600 mg/dL, so that at the end of the experiment, untreated diabetic mice showed high hyperglycemia compared to treated mice (TH‐D vs. TH‐D/Cana, *p* < 0.0001). The drug also improved glycemia in pre‐diabetic TH‐Ctrl mice (–33%) after 2 weeks of treatment but had minimal effects later (Figure 1C). In a fasting‐refeeding experiment, TH‐D mice showed severe hyperglycemia even after 16 h fasting, but Cana treatment reduced it by ~51% (*p* < 0.0001). Post‐feeding, TH‐D mice displayed severely elevated BG, while those on the Cana diet had significantly lower BG levels (*p* < 0.0001) but were still high compared to TH‐Ctrl (Figure 1D). As expected, Canagliflozin increased glucosuria in TH‐D mice (*p* < 0.001) compared to chow‐fed mice (Figure 1E). This led to significant osmotic diuresis with urine output increasing ~4 times (*p* < 0.0001) in TH‐D/Cana mice versus those on regular chow (Figure 1F).

### 3.2 Canagliflozin Improved In Vivo β‐Cell Function and Glucose Homeostasis but Didn't Change the Peripheral Insulin Resistance in TH Mice

To assess Canagliflozin's effects on glucose handling in TH mice, we fasted the mice for ~16 h and performed an intraperitoneal glucose tolerance test (ipGTT) on 19‐week‐old conscious mice. TH mice (both pre‐diabetic and diabetic) were glucose intolerant compared to the control ND group; TH‐Ctrl mice showed significant glucose intolerance (*p* < 0.01), and Canagliflozin did not significantly improve it (Figure 2A,B). However, Canagliflozin reduced the glucose excursion curve in TH‐D mice (*p* < 0.01), though levels remained elevated compared to TH‐Ctrl. As anticipated, plasma insulin levels were low in TH‐D mice (AUC_Ins_: 48.53 ± 8.97), but TH‐D/Cana mice showed 2‐times higher insulin output during the test (*p* < 0.0001) (Figure 2C,D). HOMA‐IR analysis indicated no significant baseline insulin resistance differences between groups, but HOMA‐B index showed improved β‐cell function in TH‐D mice after 10 weeks of Canagliflozin treatment (*p* < 0.05) (Figure 2E,F). Additionally, basal insulin levels and C‐Peptide (a more reliable indicator of β‐cell secretory function) were significantly elevated in TH‐D/Cana mice compared to untreated TH‐D group (Figure 2G). Glucagon levels were higher in the plasma of TH‐D mice compared to TH‐Ctrl (*p* < 0.05), but showed no significant changes with Canagliflozin treatment in either group (Figure 2H).

### 3.3 Canagliflozin Restored and Normalised Islet Morphometry in TH Diabetic Mice

**FIGURE 3:**
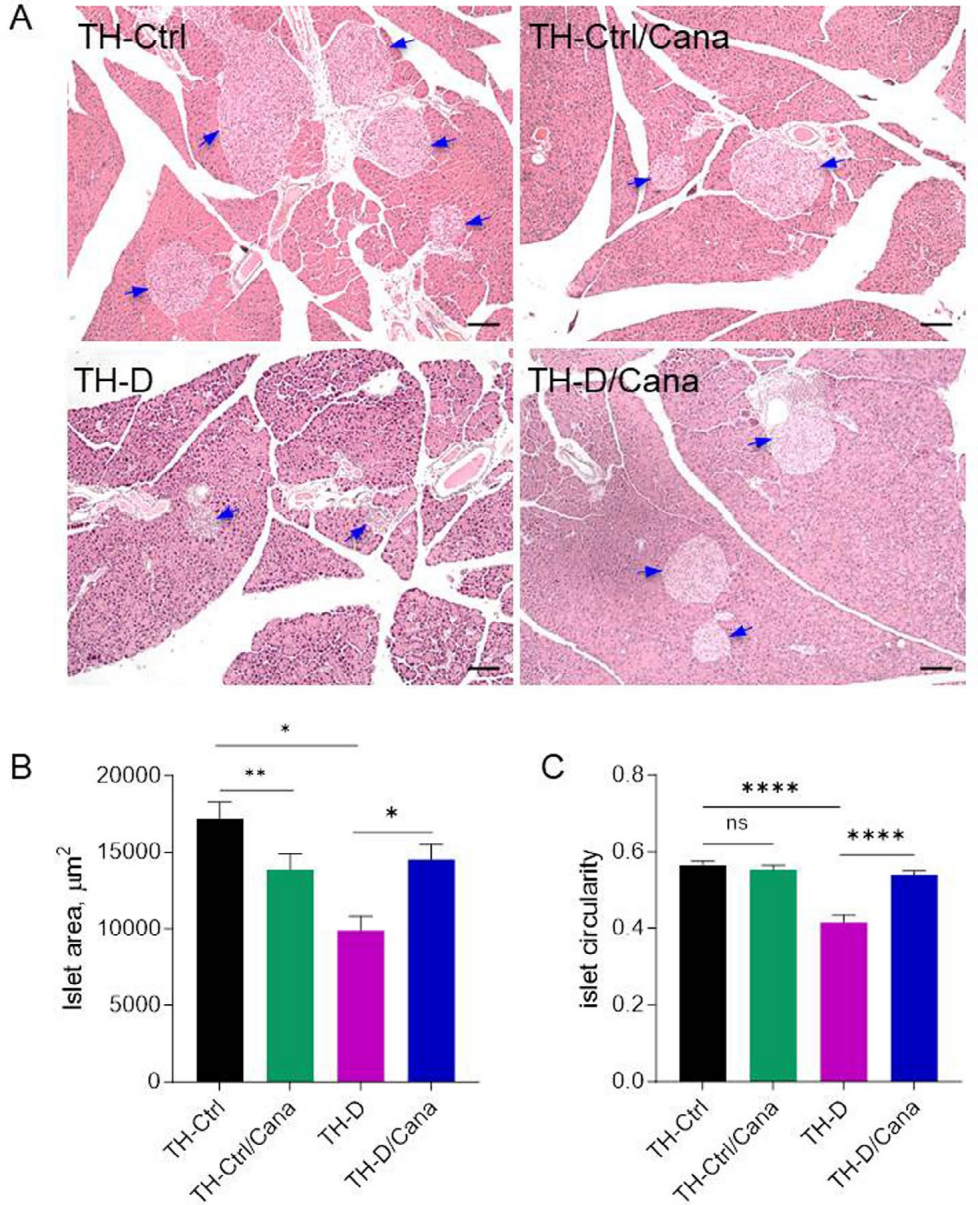
Islet histomorphometry. (A) Representative micrographs of islets (indicated with blue arrows) from H&E stained sections. Scale bar = 100 μm. (B) Mean islet area (μm^2^) and (C) islet circularity index were assessed for each experimental group on a minimum of 100 islets/group pooled from *n* = 3 mice/group and 3–4 sections/pancreas (100 μm apart). A circularity index of 1.0 indicates a perfect circle. As the value approaches 0.0, it means an increasingly elongated polygon. Data are expressed as means ± SEM. One‐way ANOVA with the Kruskal–Wallis test was applied for between‐groups analysis. *p < 0.05, **p < 0.01, ***p < 0.001, ****p < 0.0001. “ns” means not significant, ####*p* < 0.0001.

In situ morphometric analysis of pancreatic islets confirmed the deleterious effect of overt diabetes on β‐cell mass (Figure 3). In the TH pancreata, early onset diabetes significantly affected the islet average area and disrupted the shape (‘circularity’) of the islets, an indirect indicator of their health [16]. Cana demonstrated a beneficial effect on both these parameters when administered to diabetic animals [TH‐D vs. TH‐D/Cana; *p* < 0.05 (islet area); *p* < 0.0001 (islet circularity)] (Figure 3A–C); a significant change of islets' size was also observed in pre‐diabetic TH‐Ctrl mice fed with Cana diet vs. chow diet, consistent with the reduction of β‐cell hyperplasia specific to pre‐diabetic animals (TH‐Ctrl/Cana vs. TH‐Ctrl; *p* < 0.01). The pancreatic phenotype of TH‐Ctrl/Cana mice was not further investigated in the present study.

### 3.4 Canagliflozin Reduced α‐Cell Hyperplasia, Reversed the β to α‐Cell Ratio, and Restored Pancreatic Insulin Content in TH Diabetic Mice

**FIGURE 4:**
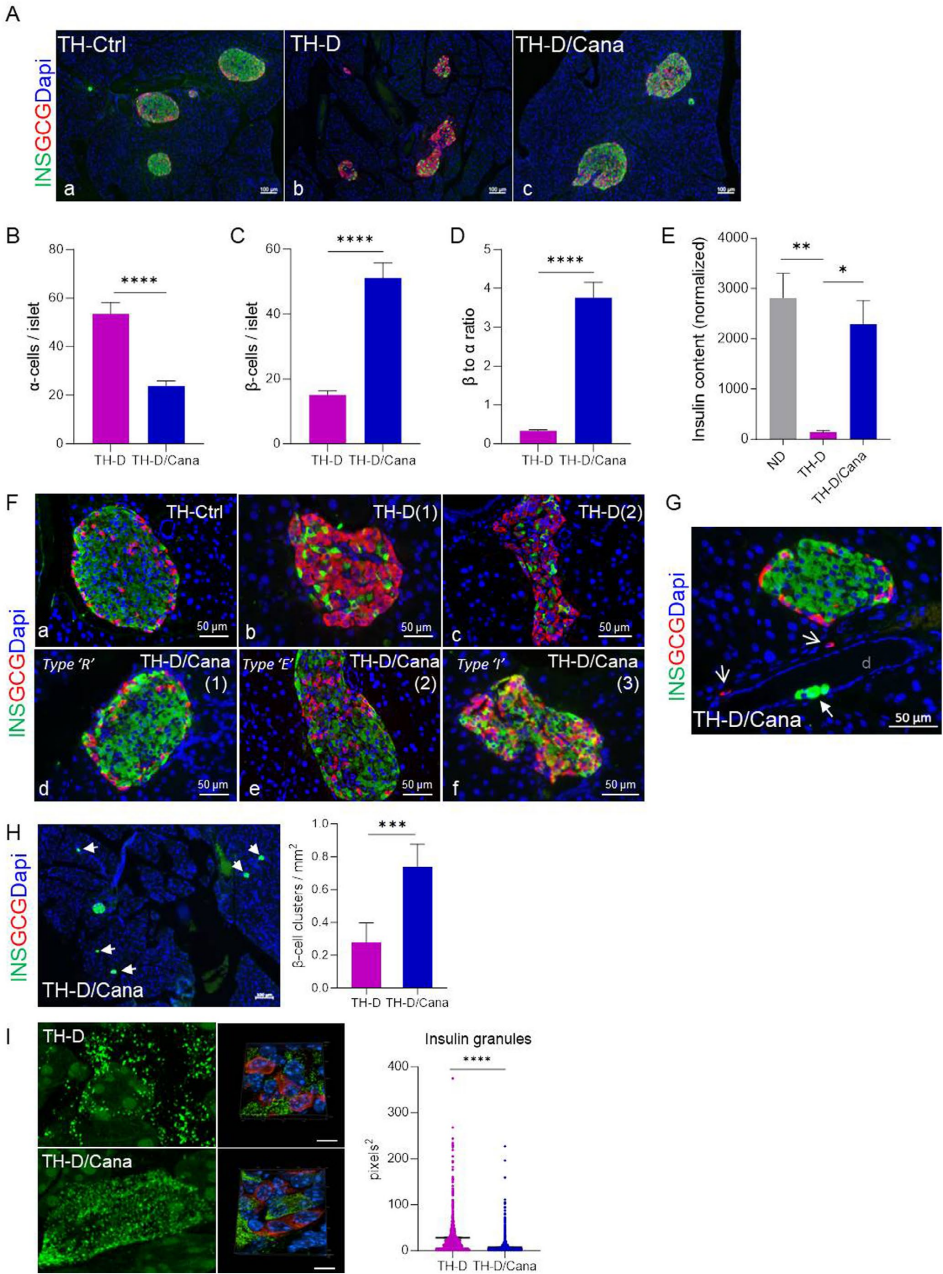
Impact of canagliflozin treatment on islet cell composition and fate. (A) Representative pictures of islets in double immunofluorescence labeling for glucagon (GCG) and insulin (INS) in pancreatic sections (*n* = 3 mice/each group). (B–D) Quantification of α and β‐cells in the islets of TH‐D and TH‐D/Cana pancreata (minimum 90 islets from *n* = 3 mice/group were counted). The bar graphs show the average number of α‐cells (B) and β‐cell (C) per islet and the β‐to‐α‐cell ratio per pancreatic cross‐section (D). Data are expressed as means ± SEM. Mann–Whitney test (two‐tailed) was applied to calculate the statistical significance. ****p < 0.0001. (E) Assessment of pancreatic insulin content normalised to total protein content in pancreatic extracts of TH‐D and TH‐D/Cana mice compared to ND mice (means ± SEM; *n* = 6 pancreata/group). (F) Details of islet morphology; (a) the round hypertrophic islets specific to TH‐Ctrl tissues; (b, c) examples of GCG+ rich islets in TH‐D pancreata; (d–f) representative pictures showing the main patterns of islet architecture identified in the TH‐D/Cana pancreata: the mantled rounded architecture (the ‘R’ type), the elongated islets (the ‘E’ type) and the irregular islets (the ‘I’ type) containing bihormonal INS+GCG+ cells (yellow) at the periphery. (G) GCG+ cells (open arrow) identified by immunostaining in the periductal compartment of the TH‐D/Cana pancreas; intraductal INS+ cluster (solid arrow). (H) Small clusters of β‐cells (≤ 5 INS+ cells) were identified in the TH‐D/Cana pancreata (white arrows); the number of clusters normalised to tissue area (mm^2^) in the TH‐D/Cana compared to the TH‐D pancreas sections. Mann–Whitney test (two‐tailed) was applied to calculate the statistical significance (*p* = 0.0003). (I) Super‐resolution microscopy images (3D and details) of the pancreas sections documenting significant differences in the morphology and the area (pixels^2^) of the cytoplasmic insulin granules of INS^+^ cells in Cana‐treated vs. non‐treated diabetic animals (area of ~1500 granules for each group was measured with ImageJ1.52a software). Scale bar = 20 μm. *p < 0.05, **p < 0.01, ***p < 0.001.

To analyse the impact of Cana on islet cell composition, we performed immunofluorescence staining of pancreas sections for the major islet hormones: insulin (INS), glucagon (GCG), and somatostatin (SST). In chow‐fed TH‐D mice, we observed smaller, irregular islets and α‐cell hyperplasia, with many GCG+ cells infiltrating the islets. Cana treatment significantly reduced GCG+ cell counts and reversed α‐cell hyperplasia (*p* < 0.0001), while remarkably increasing INS+ cell number and improving the β to α cell ratio (Figure 4A–D). The proportion of GCG+ cells in TH‐D islets (53.5 ± 4.6) closely matched the INS+ cell proportion in TH‐D/Cana islets (51.2 ± 4.5). This result was in line with the substantial augmentation of the pancreas insulin content in the TH‐D/Cana vs. TH‐D mice (Figure 4E). We identified three islet architectural patterns in TH‐D/Cana pancreata: a healthy regular mantled ‘R’ pattern with core—clustered INS+ cells (71%), an elongated ‘E’ pattern with scattered GCG+ cells (16.5%), and an irregular form ‘I’ type of which some contained bihormonal cells (INS^+^GCG^+^) at the islet periphery (12.4%), indicating an intermediary α/β phenotype (Figure 4F). A similar pattern was observed when islets were stained for GCG and SST (data not shown). Interestingly, GCG‐labelled cells were detected in the periductal compartment close to islets in several TH‐D/Cana pancreata but were absent in TH‐D sections, suggesting duct‐lining precursors may adopt an α‐cell identity under treatment (Figure 4G). We detected many small clusters of INS^+^ cells in the TH‐D/Cana compared to the TH‐D sections (24.5% vs. 8.9%), which depicts a possible β‐cell neogenesis under Cana treatment (Figure 4H). In SIM experiments, TH‐D/Cana β‐cells exhibited significantly smaller insulin granules than those of diabetic TH‐D cells (Figure 4I), indicating less β‐cell dysfunction [17].

### 3.5 Canagliflozin Did Not Induce β‐Cell Proliferation but Prevented Apoptosis in the Islets of Diabetic Mice

**FIGURE 5:**
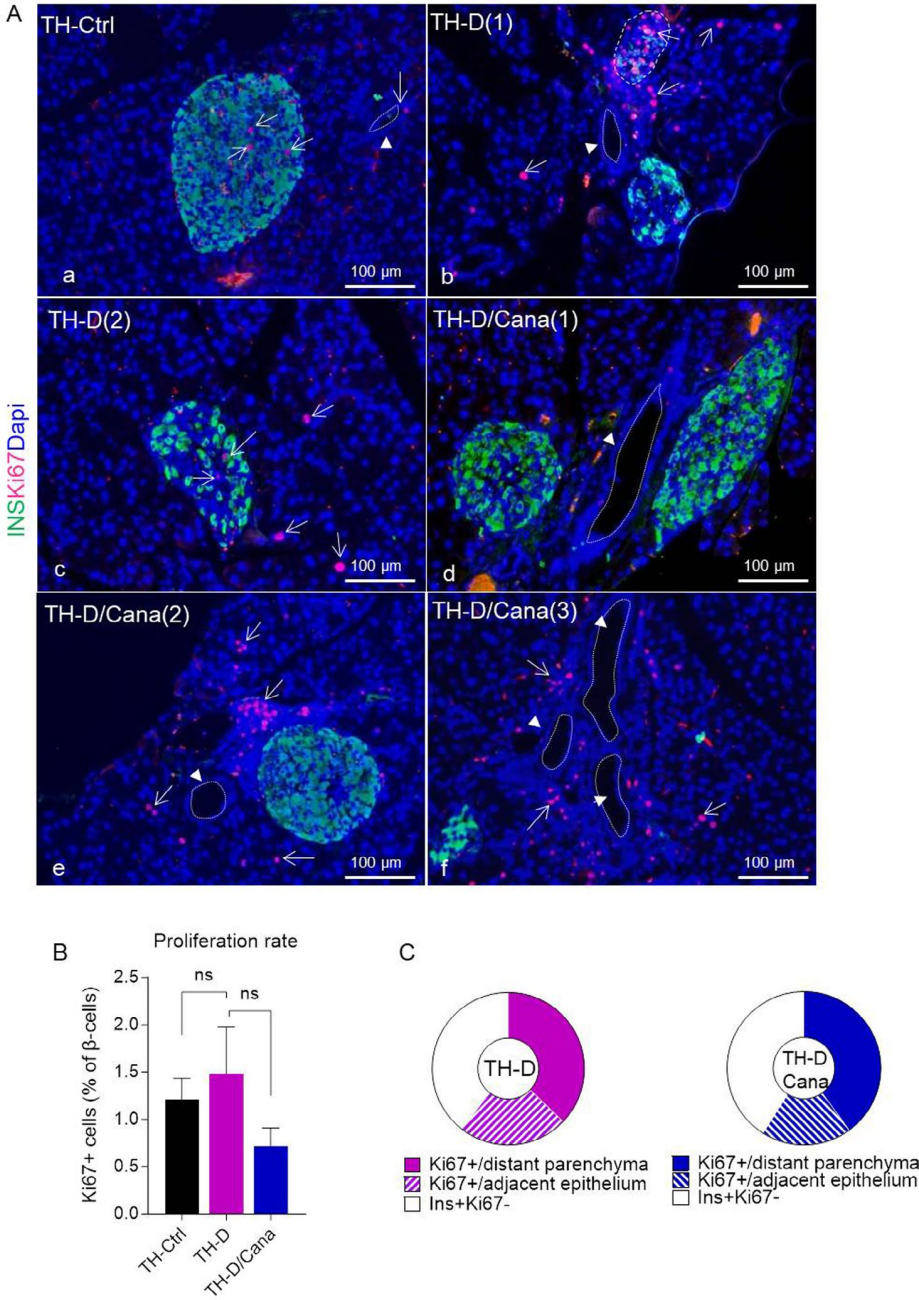
Effects of Canagliflozin on β‐cell proliferation. (A) Double immunofluorescence labeling using anti‐insulin (green) and anti‐Ki67 (red) antibodies, with DAPI counterstaining (blue) in pancreatic sections of chow‐ and Cana‐fed mice. Arrows indicate Ki67^+^ cells. Arrowheads indicate the pancreatic ducts. Few Ki67^+^Ins^+^ cells were observed in the TH‐Ctrl islets (a); visibly damaged islets displaying a few low insulin‐expressing cells contained Ki67^+^ cells (b), while undamaged diabetic islets displayed scarce Ki67^+^Ins^+^ cells and rare Ki67^+^ nuclei in the distant parenchyma (c). TH‐D/Cana section containing no intraislet Ki67+ cells (d) or multiple Ki67^+^ nuclei outside of the islets—in the parenchyma and/or in the islet or duct proximity (e, f). (B) Quantification of β‐cell proliferation in pancreatic sections, expressed as % of Ki67^+^ cells of the total INS^+^ cells/islet. Data are expressed as means±SEM (min. 50 islets/group). Kruskal‐Wallis test with Dunn's multiple comparison test was used to analyse statistical differences between groups. (C) Distribution of Ki67^+^ staining patterns in TH‐D/Cana versus TH‐D pancreas sections: islets containing INS^+^Ki67¯ cells and no or sporadic Ki67^+^ nuclei in the islet proximity (clear white area), a compact mass of Ki67^+^ nuclei within the epithelium adjacent to the islets (hatched area); Ki67^+^ nuclei found in random cells of the distant parenchyma (solid coloured area). The *χ* ^2^ test was applied to calculate the statistical significance between these groups (*p* = 0.84, not significant).

**FIGURE 6:**
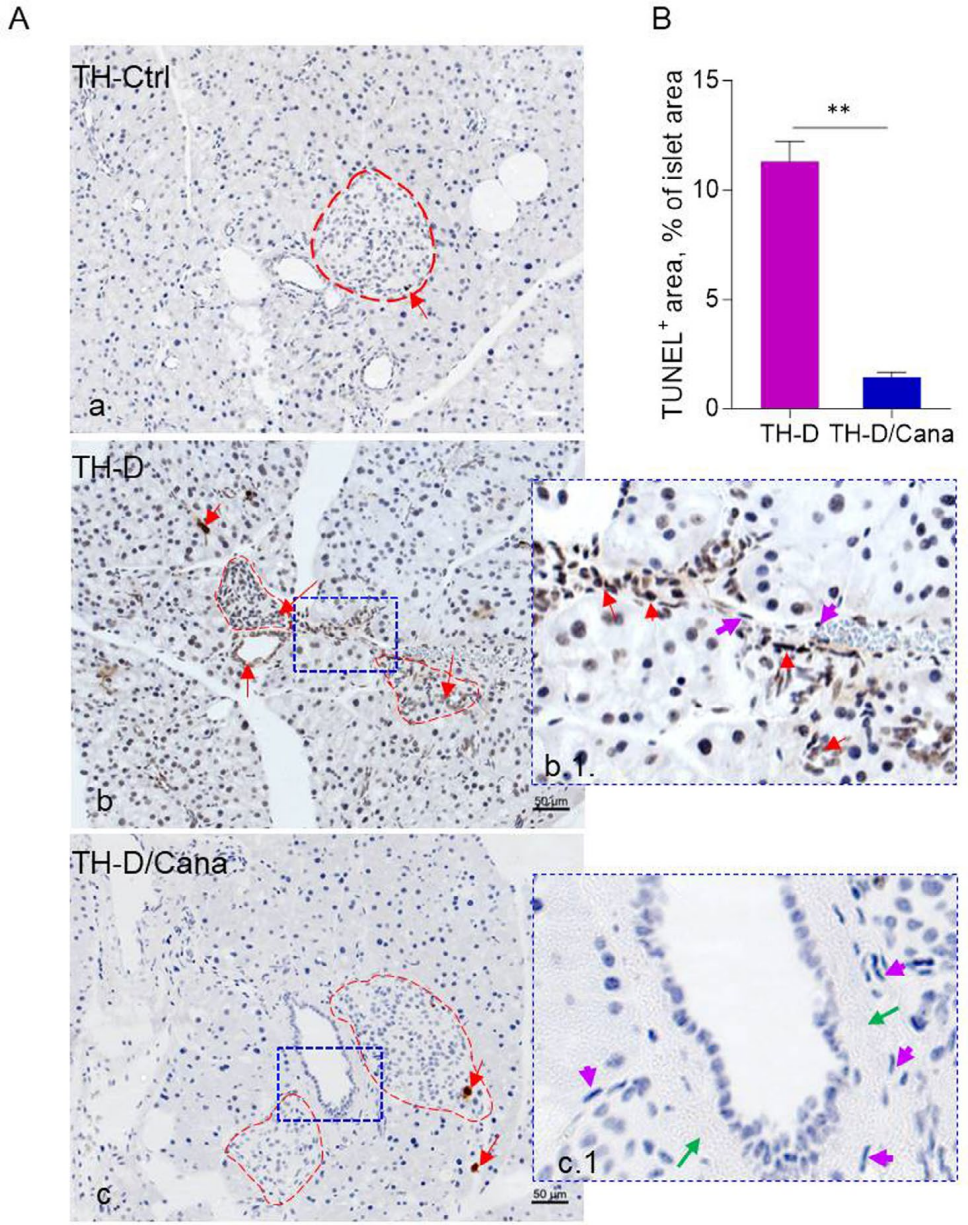
Apoptosis assessment. (A) Images of pancreatic sections of the TH‐D/Cana and TH‐D mice after TUNEL staining. Dark brown nuclei are TUNEL^+^ apoptotic nuclei (red arrows). Islets are delimited with a red dashed line (a, b, c). Magnified views of the dashed blue border areas are presented in b.1 and c.1; thickening of the ductal lining (green arrows) in the TH‐D/Cana sections containing flat nuclei (cyan arrow, c.1). (B) TUNEL^+^ area (depicting apoptosis) is expressed as % of the total islet area. Data are expressed as means ± SEM (*n* = 6–7). Mann–Whitney *t*‐test (two‐tailed) was applied to calculate the statistical significance. **p < 0.01.

To investigate whether the elevated β‐mass in TH‐D/Cana islets resulted from the self‐replication of pre‐existing β‐cells, we co‐stained the islets for INS alongside the nuclear proliferation marker Ki67. As expected, Ki67+ cells were rare in TH‐Ctrl islets (1.2% ± 0.22% of all INS+ cells) (Figure 5A‐a). In TH‐D pancreata, we observed clusters of Ki67+ nuclei either inside the heavily damaged islets (in INS^−^ cells) or lining the ducts or in the adjacent epithelium (Figure 5A‐b,c). TH‐D/Cana islets contained ~41% INS^+^Ki67¯ cells with sporadic Ki67^+^ nuclei nearby, while 19% were well‐defined islets (in the proximity of a duct) together with an adjacent dense mass of small round cells of which a part was actively proliferating (Figure 5A‐d,e). Ki67^+^ nuclei were also found in random cells of the distant parenchyma and in the epithelium lining the ductal lumen (40% of total counted islets) (Figure 5A‐e,f). Notably, GCG+ cells in both groups were negative for Ki67, indicating no self‐replication of α‐cells either. Overall, the amount of proliferating β‐cells was low, no significant differences were obtained between our experimental groups, and proliferation is unlikely to explain β‐cell replenishment in the Cana‐treated diabetic mice (Figure 5B,C). Finally, Cana treatment significantly prevented apoptosis, extensively detected in the untreated TH‐D islets (*p* < 0.01) and nearby cells; many non‐apoptotic flat nuclei (TUNEL^Neg^), reminiscent of mesenchymal cells, were noted in the thicker linings of TH‐D/Cana ducts (Figure 6).

### 3.6 Canagliflozin Restored and Stabilised β‐Cell Identity in TH Islets

**FIGURE 7:**
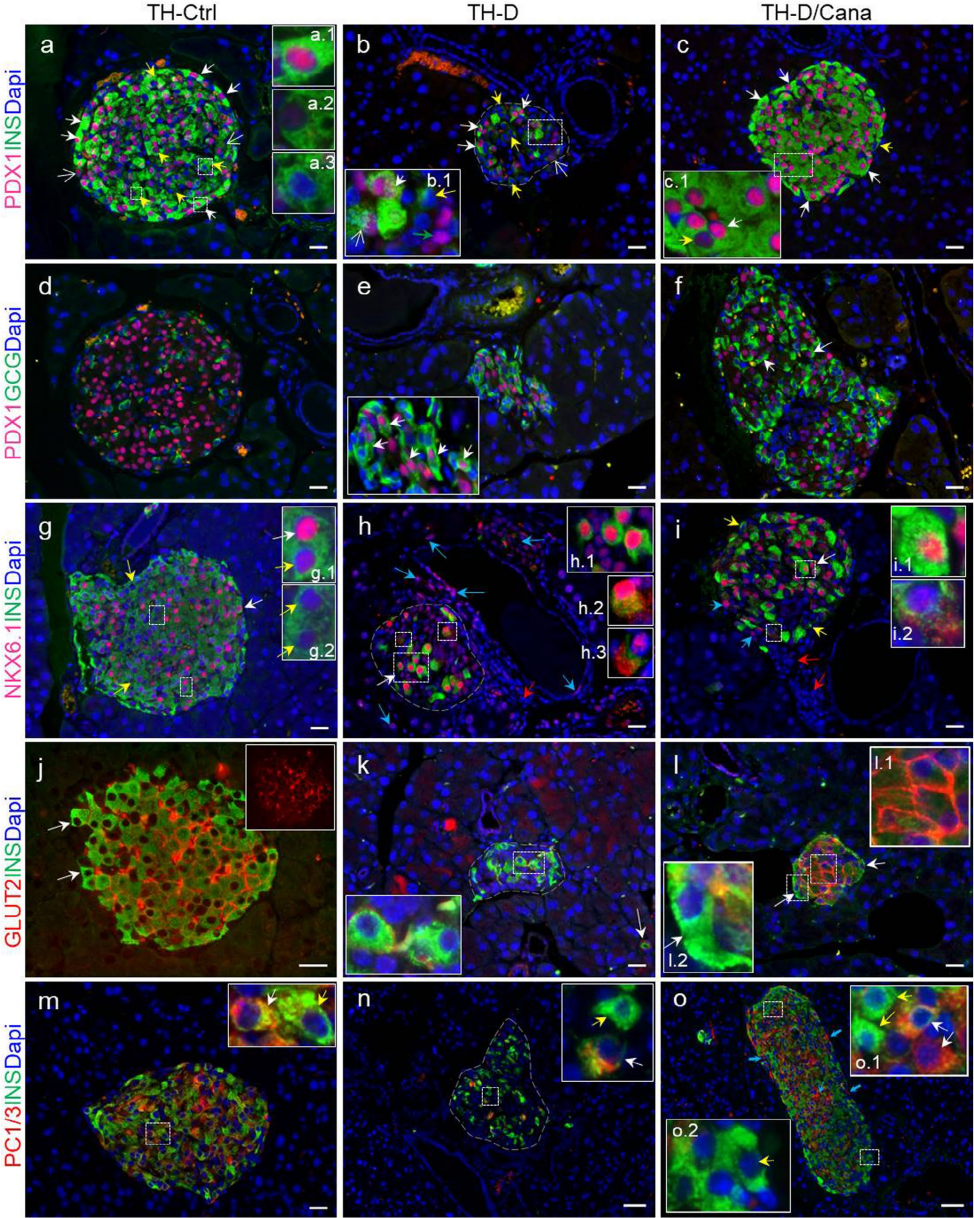
Expression of several bona fide β‐cell markers detected by immunofluorescence in the pancreas sections from TH‐Ctrl, TH‐D and TH‐D/Cana mice using the indicated antibody combinations. Magnified views of the white dashed border rectangles are presented in insets. (a–c) PDX1 transcription factor is expressed in the nuclei of INS^+^ cells at different intensities; white solid arrow = PDX^High^INS^+/High^ cells (a.1); white open arrow = PDX1^+^ INS^Low^ cells; yellow solid arrow = PDX1^Low/Neg^INS^+^ cells (a.2; a.3; c.1); green solid arrow = PDX1^+^INS^Neg^ cells (b.1 inset). (d–f) Immunolabelling of GCG and PDX1 in pancreatic sections. GCG^+^ cells do not express the nuclear factor PDX1, which is limited to β‐cells in TH‐Ctrl islets (d). Abnormal PDX1^+^GCG^+^cells (white arrows) were observed in many TH‐D islets (e). In the sections of the TH‐D/Cana pancreata, most of the GCG‐expressing cells were negative for PDX1 (f); only rare PDX1^+^GCG^+^ cells were found in the ‘I’ or ‘E’‐type islets (white arrows). (g–i) NKX6.1 expressed with different intensities in the nuclei: white solid arrow = NKX6.1^High^INS^+/High^ (g.1, h.1, i.1); yellow solid arrow = NKX6.1^Low/Neg^INS^+^ cells (g.2); NKX6.1 localized in both the nucleus and the cytosol (h.2, h.3, i.2); the blue solid arrows depict cells of the ducts or located in the epithelium lining the ductal lumen or within the proximal exocrine tissue displaying transient nuclear NKX6.1. Agglomerations of small NKX6.1^Neg^ nuclei in the epithelium adjacent to islets or to the pancreatic ducts were very visible in (h) and (i) (red arrows). (j–l) Expression of the GLUT2 transporter in the TH‐Ctrl islets. For clarity, only GLUT2 staining is shown in the inset (j). GLUT2 was absent from the membrane of residual TH‐D β‐cells (k) but was re‐expressed in β‐cell membrane after Cana treatment (l.1). GLUT2^Neg^INS^+^ cells located at the edge of the recovered islets are indicated with a white solid arrow (l.2). (m–o) Detection of the cytosolic expression of PC1/3 in β‐cells. INS^+^PC1/3^+^ cells (white arrows) and scattered INS^+^PC1/3^Neg^ cells (yellow arrows) were identified in TH‐Ctrl islets (m). The PC1/3 expression was lost in most of the residual β‐cells of the TH‐D islets (n) but was re‐expressed in a larger population of INS^+^ cells upon Cana treatment (o). However, INS^+^PC1/3^Neg^ cells were still detected in many elongated islets toward the pole of periductal cellular accumulations (o.1, o.2). Blue arrows depict PC1/3^+^INS^Neg^ cells. Scale bar = 20 μm (a–m). Scale bar = 50 μm (n,o).

To further assess the phenotype and subsequently the functionality of the β‐cells in the Cana‐treated mice, we used fluorescent‐labelled antibodies to stain several transcription factors important for the terminal maturation of β‐cells. This experiment revealed a high heterogeneity of β‐cells within the same TH animal, similar to humans. The canonical PDX1 (pancreatic/duodenal homeobox 1), necessary for the pancreatic epithelium specification during development and a marker of adult β‐cells, was restricted to INS^+^ cells in the control TH mice, at different fluorescent intensities: PDX1^Low/Neg^INS^+^ cells, abundant toward the core of TH‐Ctrl islets, PDX1^High^INS^High^ cells present mostly toward the edge (Figure 7a); the unwound diabetic islets displayed a few PDX1^High^INS^High^ cells and PDX1^Low/Neg^ INS^+/Low^cells (Figure 7b) depicting a β‐cell identity loss. Conversely, the ‘R‐type’ TH‐D/Cana islets contained many PDX1^High^INS^High^ cells (Figure 7c), while the elongated ‘E‐type’ islets preponderantly had scattered PDX1^+^INS^Low/Neg^ and PDX1^Neg^INS^+^ cells, suggesting that these islets still contain immature or de‐differentiated β‐cells (data not shown). Many TH‐D islets contained abnormal PDX1^+^GCG^+^ cells (23.4% ± 0.3% of total GCG^+^ cells/islet), indicative of a changing phenotype (β‐cell losing identity or progenitors acquiring an α fate) [18, 19], while fewer PDX1^+^GCG^+^ cells (7.8% ± 0.5% of total GCG^+^ cells/islet) were found in the sections of the TH‐D/Cana pancreata, indicative of a stabilised α or β phenotype (Figure 7d,e). This suggests that Cana treatment favoured the expression of PDX1 within the existing or recovered β‐cells, stabilising their β‐phenotype.

The NKX6.1 transcription factor, essential for β‐cell identity, development and function [20], was also expressed at variable levels in the nuclei of INS^+^ cells. Most of the INS^+^ cells of TH‐Ctrl mice expressed correctly the nuclear NKX6.1 but others were negative or contained low NKX6.1 expression (Figure 7g). Nuclear NKX6.1 was preserved in a few surviving β‐cells together with INS (NKX6.1^Hig h^INS^High^) in TH‐D islets, while it was localised in both the nucleus and the cytosol of other INS^+^ (β‐cells undergoing dedifferentiation) or was expressed in the nuclei of other cells of the islets (Figure 7h). In a few cases, cells of the ducts or located in the epithelium lining the ductal lumen or within the proximal exocrine tissue displayed the nuclear NKX6.1 marker (Figure 7h). This extra‐islet localization of NKX6.1 was absent in the Cana‐treated diabetic islets (Figure 7i): NKX6.1 expression was restricted to INS^High^ cells mixed with a little mass of β‐cells with low insulin content, a part of which were undergoing NKX6.1 migration to the cytosol (Figure 7i). Altogether, these data show that Cana restored the expression of NKX6.1 in INS^+^ cells, fostering the β‐cell phenotype.

The β‐cell specific glucose transporter GLUT2 was absent from the membrane of residual TH‐D β‐cells but was restored in most of INS^+^ cells upon Cana treatment (Figure 7j–l); however, many INS^+^ cells from the edge of the recovered islets lacked GLUT2, similar to those of TH‐Ctrl islets. Whether these cells indicate functionally immature β‐cells or a true neogenic niche of newly formed islets [21] remains to be established.

Additionally, Cana partially restored the cytosolic expression of the prohormone convertase 1/3 (PC1/3) in diabetic animals (Figure 7m–o). Islets of untreated TH‐D mice were almost totally depleted of this critical enzyme for proinsulin processing; the analysis of the TH‐D/Cana islets stained for INS and PC1/3 (which normally co‐localises with INS in β‐cells), evidenced the presence of many PC1/3^+^INS^+^ cells but also PC1/3^Neg^INS^+^ cells, indicating that a subset of β‐cells are immature. Few cells stained only for PC1/3 that might be related to the expression of the convertase in non‐β‐cells (potential pro‐α‐cells) [22]. TH‐Ctrl pancreata also contained PC1/3^Neg^INS^+^ cells scattered through PC1/3^+^INS^+^ cells, suggesting that dedifferentiated or immature β‐cells are also present at the pre‐diabetic stage in TH mice.

In TH‐D islets, many duct‐lining cells adopted a pro‐endocrine identity through the re‐expression of the NGN3 transcription factor (Figure S1a,b), typically absent in adult ductal cells [23]. NGN3^+^INS^Low^ cells were found in the damaged TH‐D islets, potentially suggesting a β‐cell reversion to a pre‐β state under severe hyperglycemia [23]. Notably, fewer NGN3^+^ precursors were observed in the recovered TH‐D/Cana islets compared to TH‐D islets. Whether Cana drives the differentiation of the newly formed β‐cells from NGN3+ precursors in TH‐D/Cana islets remains to be determined.

The precursors near the duct lining and cell‐dense clusters in TH‐D and TH‐D/Cana islets re‐expressed the mesenchymal marker vimentin (VIM) (Figure S1c,d). Many residual β‐cells of TH‐D islets were positive for both INS and VIM, indicating a reactivation of the EMT (epithelial‐mesenchymal transition) program during dedifferentiation in diabetes [24, 25]. In TH‐D/Cana islets, VIM^+^INS^Neg^ cells were found scattered within the islets and in the mantle surrounding them; but in larger TH‐D/Cana islets, VIM^+^ cells were limited to extra‐islet cell clusters near the ducts. Altogether, these findings suggest that Cana might promote islet repair and regeneration in diabetic mice by completing the endocrine developmental program triggered by hyperglycemia and β‐cell injury.

## 4 Discussion

An important shortage of pancreatic β‐cells is the primary cause of type 1 diabetes (T1D), while patients with T2D experience progressive β‐cell loss. Therefore, targeted therapies aimed at rescuing or regenerating β‐cells from endogenous sources represent an attractive alternative for diabetes treatment, especially if such therapies can be achieved using safe bio‐pharmacological agents. In this study, we demonstrate that Canagliflozin has the potential to recover functional β‐cell mass in the damaged islets of diabetic TH mice, a polygenic mouse model of T2D that resembles a severe form of the disease encountered in children and adolescents. Indeed, the progression of diabetes is more aggressive at younger ages [26], with a rapid decline in β‐cell function (~20% per year), compared to a slower decline (~7% per year) in adult‐onset diabetes [3]. In contrast, the ‘honeymoon phase’—a temporary remission of the disease following the initiation of insulin treatment—is more prevalent in post‐puberty and some young adults with T1D [27]. This suggests a potential recovery window for remaining β‐cells and/or an increase in β‐cell reserve from endogenous sources within that frame time [28]. Given this evidence, the paediatric TallyHo model is advantageous for studying the rapid decline in β‐cell function and the possible recovery window under pharmacological interventions. Our previous research indicates that plasma C‐peptide levels are significantly decreased in TH mice by 7 weeks of age and have already become blunted by 11 weeks of age [14]. This decline is accompanied by a near‐total loss of the β‐cell GLUT2 transporter and other alterations in β‐cell‐specific differentiation markers, which indicates an early loss of β‐cell identity and function in these animals [14]. However, our model does not reflect a near‐total β‐cell destruction, as is achieved with a high‐dose of STZ, for example [29], or with another ablation method [10, 11]. In the present study, treatment with Canagliflozin commenced when the mice were 10 weeks old, which falls within a period of β‐cell impairment and identity loss. The 10‐week Canagliflozin treatment improved β‐cell function, as measured by the HOMA‐B index, and was confirmed by increased levels of C‐peptide and insulin in diabetic mice compared with the un‐treated mice, along with the re‐expression of GLUT2 in most INS+ cells. In prediabetic TH‐Ctrl mice, Canagliflozin reduced basal C‐peptide levels, likely due to a smaller β‐cell mass reflected in smaller islet size (Figure 3B), but this was not necessarily or sufficiently correlated with changes in blood glucose levels or insulin resistance, as indicated by the unchanged HOMA‐IR index in these mice.

The improved β‐cell function in diabetic TH mice by Cana treatment was paralleled by a significant recovery of β‐cell mass and islet reconstruction. Given that β‐cell proliferation is not increased in TH‐D/Cana pancreata and that reduced apoptosis alone cannot explain the magnitude of β‐cell mass recovery (Section 3.5), the cellular source of newly formed or restored β‐cells remains a central unresolved question in this model. Our histological and molecular analyses therefore suggest that several non‐mutually exclusive mechanisms may contribute to this process, including trans‐differentiation from α or non‐endocrine pancreatic cells, differentiation from endocrine progenitors, and recovery of dedifferentiated β‐cells. Our study's evidences supporting each mechanism are discussed below.

The significant increase in INS^+^ cell mass following canagliflozin treatment in severely diabetic mice was accompanied by a proportional decrease in GCG^+^ cell counts, suggesting a possible α‐to‐β conversion. It has been suggested that in diabetes, the α‐cell population may act as a ‘rescue reservoir’ for β‐cell neogenesis through cellular reprogramming or trans‐differentiation in adult animals [30]. This is supported by studies showing that spontaneous trans‐differentiation of α to β‐like cells can occur under conditions of extreme β‐cell loss [10] or with specific bio‐pharmacological interventions [31, 32], although other studies have contradicted this hypothesis [33, 34]. In our model, we observed rare bi‐hormonal INS^+^GCG^+^ cells, which, again, could indicate an ongoing trans‐differentiation process in the irregularly shaped islets (richer in GCG^+^ cells) of TH‐D/Cana mice, where the formation of new β‐cell‐like structures is still incomplete. A similar conversion process has been reported in two other T2D murine models treated with dapagliflozin for 6 weeks [35], although this was not observed in STZ or high‐fat diet‐induced diabetes treated with the same drug over a period of 10–12 days [36]. The drug doses used in these studies were similar, but we believe that the discrepancies arise from the treatment duration (the longer, the better), the model of β‐cell deficiency, and the timing of gliflozin administration (earlier is better), as other authors also suggested [37, 38]. On the other hand, the presence of bi‐hormonal INS^+^GCG^+^ cells can solely be attributed to an intermediary state between an α and a β phenotype, as a result of the dedifferentiation process or abnormal cell maturation. Therefore, further investigations using lineage‐tracing models and appropriate translational validation studies in human pancreata (where bi‐hormonal INS^+^GCG^+^ cells have also been observed [18]) are necessary to confirm the potential of cana to trigger α‐to‐β transdifferentiation.

In TH‐D pancreata, we observed a significant mobilisation of epithelial cells near the ductal epithelium that adopted an endocrine cell identity. This change occurred through the re‐expression of the pro‐endocrine NGN3 transcription factor (typically active during embryonic development), as a result of islet damage [39]. However, in many sections of TH‐D/Cana pancreas, the re‐expression of NGN3 was less abundant than in TH‐D samples. This suggests that Cana favours the maturation and stabilisation of newly specified endocrine cells rather than the activation of the NGN3 progenitors (which is triggered by β‐cell injury and loss). An interesting observation was the presence of GCG+ cells in the periductal compartment of the TH‐D/Cana pancreas, indicating that Cana may support the specification of α‐cells that further undergo transdifferentiation to β‐cells. A direct conversion from NGN3+ progenitors to β‐cells is unlikely in this context but cannot be totally excluded since small INS^+^ clusters were present in TH‐D/Cana pancreas parenchyma and newly formed β‐cells can originate directly from acinar NGN3‐expressing cells or other potential precursors [40].

In parallel, we observed extensive clusters of VIM^+^ cells located at the site of residual TH‐D islets and within the periductal space. The reactivation of EMT canonical markers like vimentin in β‐cells has been associated with β‐cell identity loss in other models of β‐cell dedifferentiation [24, 25, 29] and is considered a regenerative compensatory response to injury. However, a full regeneration process does not occur spontaneously in damaged diabetic islets.

We demonstrated that Cana preserved the expression of PDX1, a key β‐cell transcription factor that represses the execution of the EMT program [25]. This can explain why well‐defined recovered TH‐D/Cana islets do not contain VIM^+^INS^+^ cells: Cana reestablishes the expression and transcriptional activity of PDX1 in β‐cells, preventing their transition from an epithelial to a mesenchymal phenotype. In other words, Cana favours the differentiated state of β‐cells and counteracts the EMT reactivation and loss of β‐identity. All results related to β‐cell‐specific transcription factors or markers, presented in Section 3.6, support this hypothesis: Cana stabilises the β‐cell phenotype and prevents (at least partially) the loss of β‐cell identity in our diabetes model. Because the re‐expression of vimentin is unlikely to occur in NGN3^+^ progenitors undergoing EMT [29], and since VIM^+^ cells were observed both inside and around the TH‐D/Cana islets that have not yet fully recovered, we believe that the source of VIM+ cells in TH‐D/Cana islets are either still dedifferentiated β‐cells undergoing redifferentiation or ductal precursors. We suspect that a subset of mesenchymal cells may migrate from the ducts into the islets to promote their repair and survival. This reminds us that the transplantation of mesenchymal stem cells (MSCs), through their complex secretomes, is a promising strategy for preserving β‐cell function and mass in type 1 diabetes [41].

Taken together, our data argue against β‐cell proliferation as the primary driver of β‐mass recovery by Cana and instead support a model involving a combination of β‐cell redifferentiation and limited neogenesis from progenitor or trans‐differentiated sources. Nevertheless, the relative contribution of each mechanism cannot be conclusively determined from the present study and warrants further investigation.

Although we did not observe an overall increase in islet size in TH‐D/Cana compared to TH‐Ctrl (and TH‐Ctrl/Cana), despite the exaggerated hypertrophy described in other studies of induced β‐cell regeneration [32, 42], we cannot rule out the possibility that a longer treatment or higher doses of Cana may lead to larger islets with abundant β‐cells, or alternatively, that islet replenishment could be limited. Additionally, an important question emerges regarding whether the newly formed islets can be maintained and remain functional when Cana treatment is discontinued and as the mice age.

The hypothesis of glucolipotoxicity alleviation by Cana cannot be excluded either. It has been suggested that the protective effect of gliflozins on β‐cells is an indirect benefit of the improved peripheral glucose tolerance and reduced hyperglycemia [43, 44], which may relieve metabolic stress and its consequences (mitochondrial ROS generation, ER stress, and apoptosis) in β‐cells [45, 46]. While current hypoglycemic agents can improve β‐cell function and preserve β‐cell mass in the long term [47] there is no evidence that they can regenerate the endogenous β‐cell reservoir. A recent study demonstrated that, despite effectively normalising glycemia in db/db mice, insulin glargine alone did not preserve β‐cell mass. In contrast, dapagliflozin, either alone or in combination with glargine, did [48].

Furthermore, a small dose of dapagliflozin, while ineffective in reducing hyperglycemia in two other diabetes‐like models, was able to restore β‐cell insulin content and mass and normalise the islet phenotype [36]. This suggests that other mechanisms, independent of glycemic control, such as the reactivation or facilitation of an endocrine regeneration program (as we propose herein), may be responsible for β‐cell maintenance by gliflozins, particularly when applied during a specific stage of the disease. Conversely, at advanced stages of β‐cell destruction, even efficient hypoglycemic action may not suffice to save or regenerate these cells.

SGLT2i influence various metabolic pathways, shifting fuel utilisation from glucose to lipids and ketones as effective energy sources, a concept known as the ‘thrifty substrate hypothesis’ [49]. Thus, protecting against lipid overload and enhancing energy metabolism in multiple tissues, including the pancreas, may indirectly contribute to the benefits of this class of anti‐diabetics.

Finally, since SGLT2 is not expressed in pancreatic β‐cells and its presence in α‐cells is controversial [9, 50], we cannot ignore a possible off‐target effect mediated by another molecular target or signalling pathway active in β‐cells, as demonstrated in other tissues [51, 52]. While canagliflozin is considered in clinical practice a selective SGLT2i, it also inhibits SGLT1 transporters (albeit with less potency) in the α‐cells of the pancreas, resulting in suppression of GCG in isolated islets [53]. Although Cana treatment did not significantly alter plasma GCG concentrations in diabetic mice, a local reduction in GCG signalling may lead to the reactivation of NGN3+ progenitors and facilitate the ongoing generation of GCG‐expressing cells [42] (Figures S2 and 4G).

Research indicates that other SGLT2i, such as dapagliflozin, empagliflozin, and luseogliflozin, may also help protect and enhance β‐cell function, potentially reversing some consequences of β‐cell dysfunction in diabetes [54]. However, the mechanisms behind these effects are not yet fully understood, and can vary significantly depending on the research model, protocol used, gliflozin molecule, or duration of the intervention. We believe that canagliflozin activates several repairing mechanisms simultaneously (Figure S2), which may intersect or occur sequentially, depending on the type and degree of islet injury, local islet resources, and systemic factors. Additional approaches such as scRNAseq, lineage‐tracing experiments (indicative for the fate and origin of islet cell subpopulations) or combinations of other maturation indicators (like UCN3, Aldh1A3) and stemness markers are necessary to better understand how canagliflozin replenishes β‐cell mass in situ, stabilises β‐cell phenotype and modulates islet cell plasticity in the context of severe early‐onset T2D.

## Author Contributions

**Iuliana Popescu:** conceptualization (lead), data curation (lead), formal analysis (lead), investigation (lead), methodology (lead), project administration (lead), writing – original draft (lead), writing – review and editing (lead). **Robert C. Bunn:** investigation (equal), methodology (equal), validation (equal), visualisationvisualization (equal). **Phil Ray:** investigation (equal), methodology (equal), validation (equal), visualisationvisualization (equal). **Kathryn M. Thrailkill:** conceptualization (supporting), formal analysis (supporting), project administration (supporting), resources (supporting), writing – review and editing (equal). **John L. Fowlkes:** conceptualization (supporting), project administration (supporting), resources (supporting), writing – review and editing (equal).

## Funding

This work was supported by the National Institutes of Health NIH grants [1R21AR070620‐01] to K.M.T. and [7R01DK084045‐04] to J.L.F. Additional funding was provided by the University of Kentucky Barnstable Brown Diabetes Center Paediatric Laboratory Endowment.

## Conflicts of Interest

The authors declare no conflicts of interest.

## Supporting information


**Figure S1:** Detection of the NGN3 and VIM in the islets of the TH‐D and TH‐D/Cana mice. (a, b) NGN3^+^INS^Low^ cells (inset a1, a2) were detected in severely deteriorated islets of TH‐D mice, while highly insulin‐expressing cells residing in entire islets do not express this marker (orange arrow); numerous NGN3^+^ precursors (white arrows) were found within the epithelial cells lining the ducts and, to a lesser extent, within the endocrine compartment. (c, d) Re‐expression of the mesenchymal marker vimentin (VIM). In TH‐D pancreata, small round‐shaped VIM^+^INS^−^ lined the ducts (c.1); VIM^+^INS^+^ cells were identified at the site of residual islets (c.2). In TH‐D/Cana pancreata, clusters of VIM^+^INS^−^ cells were identified within the epithelial lining of the ducts near the islets (d.1; white arrow), in the islet mantle (white open arrow) or within the blood vessels in the islet proximity (orange arrow), and to a lesser extent, scattered within the core of islets (d.2). For clarity, separate staining of VIM and INS are shown in c.3‐c.4, d.3‐d.4.

**Figure S2:** Putative mechanisms involved in Cana‐induced islet recovery and β‐cell regeneration in severely diabetic TH mice. Cana inhibits β‐cell dedifferentiation and the apoptosis of β‐cells and ductal progenitors. Cana stabilises the epithelial fate of β‐cells, prevents their EMT reprogramming and favours the transdifferentiation of α to β‐like cells and the replenishment of the β‐cell reservoir. The putative driving force of this process (blue arrows) is the reactivation of an endocrine developmental program favouring the transition of specific ductal precursors to NGN3^+^ endocrine progenitors and α‐like cells.

**Appendix S1:** jcmm71041‐sup‐0003‐AppendixS1.docx.

## Data Availability

The data that support the findings of this study are available from the corresponding author upon reasonable request.
