# Supplementary figures and images for: The SGLT2 Inhibitor Canagliflozin Promotes β‐Cell Regeneration and Restores and Stabilises β‐Cell Identity in a Polygenic Model of Severe Early‐Onset Type 2 Diabetes
Source: J Cell Mol Med. 2026 Mar 11;30(5):e71041. doi: 10.1111/jcmm.71041 (PMC13097495; doi:10.1111/jcmm.71041)

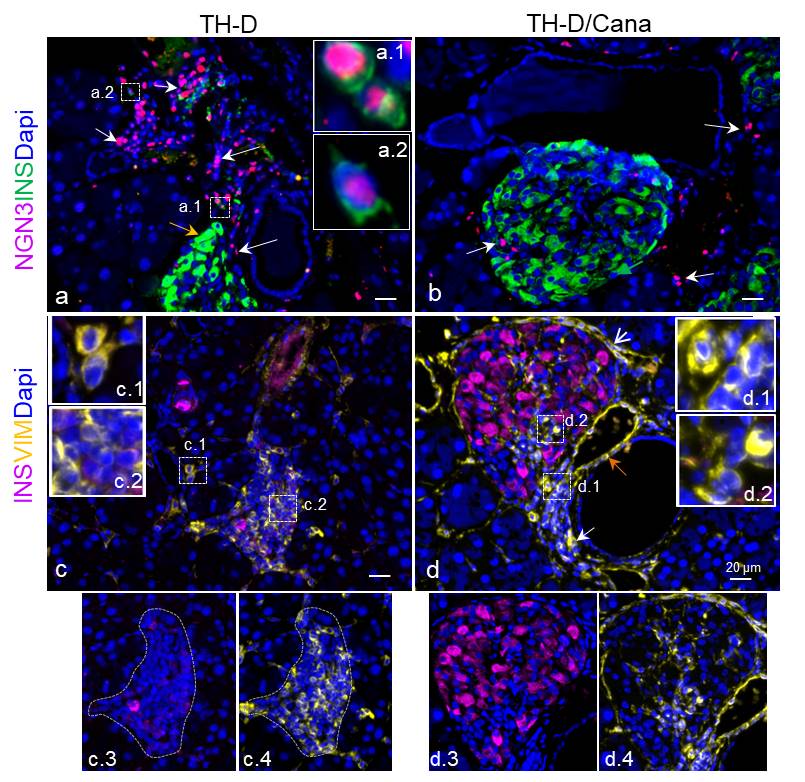

Supplement: Supplementary file 1 — Figure S1: Detection of the NGN3 and VIM in the islets of the TH‐D and TH‐D/Cana mice. (a, b) NGN3+INSLow cells (inset a1, a2) were detected in severely deteriorated islets of TH‐D mice, while highly insulin‐expressing cells residing in entire islets do not express this marker (orange arrow); numerous NGN3+ precursors (white arrows) were found within the epithelial cells lining the ducts and, to a lesser extent, within the endocrine compartment. (c, d) Re‐expression of the mesenchymal marker vimentin (VIM). In TH‐D pancreata, small round‐shaped VIM+INS− lined the ducts (c.1); VIM+INS+ cells were identified at the site of residual islets (c.2). In TH‐D/Cana pancreata, clusters of VIM+INS− cells were identified within the epithelial lining of the ducts near the islets (d.1; white arrow), in the islet mantle (white open arrow) or within the blood vessels in the islet proximity (orange arrow), and to a lesser extent, scattered within the core of islets (d.2). For clarity, separate staining of VIM and INS are shown in c.3‐c.4, d.3‐d.4. [file JCMM-30-e71041-s003.jpg]

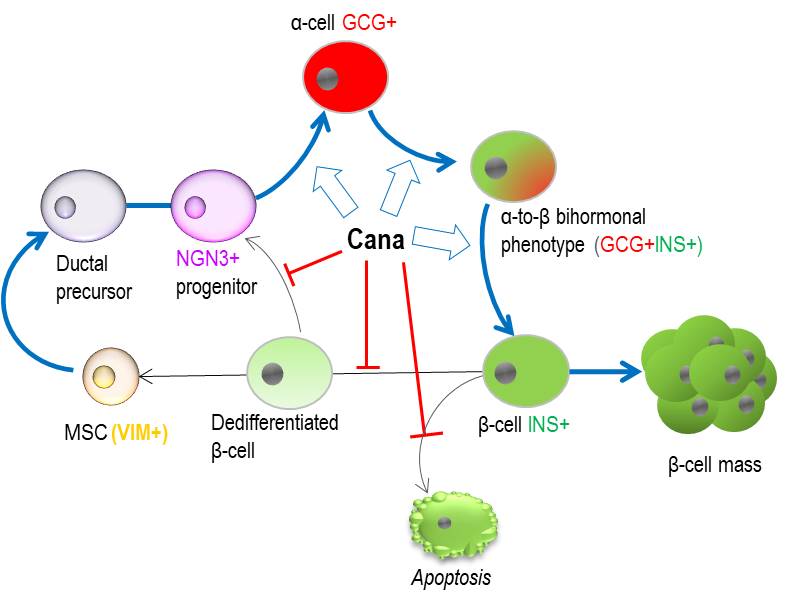

Supplement: Supplementary file 2 — Figure S2: Putative mechanisms involved in Cana‐induced islet recovery and β‐cell regeneration in severely diabetic TH mice. Cana inhibits β‐cell dedifferentiation and the apoptosis of β‐cells and ductal progenitors. Cana stabilises the epithelial fate of β‐cells, prevents their EMT reprogramming and favours the transdifferentiation of α to β‐like cells and the replenishment of the β‐cell reservoir. The putative driving force of this process (blue arrows) is the reactivation of an endocrine developmental program favouring the transition of specific ductal precursors to NGN3+ endocrine progenitors and α‐like cells. [file JCMM-30-e71041-s001.jpg]
