# Supplementary material for: The SGLT2 Inhibitor Canagliflozin Promotes β‐Cell Regeneration and Restores and Stabilises β‐Cell Identity in a Polygenic Model of Severe Early‐Onset Type 2 Diabetes
Source: J Cell Mol Med. 2026 Mar 11;30(5):e71041. doi: 10.1111/jcmm.71041 (PMC13097495; doi:10.1111/jcmm.71041)
Supplement: Supplementary file 3 — Appendix S1: jcmm71041‐sup‐0003‐AppendixS1.docx. [file JCMM-30-e71041-s002.docx]

**Animal experiments**

All animal experiments were conducted according to the *Guide for Care and Use of Laboratory Animals* (National Institutes of Health, Bethesda, MD) and were approved by the local Institutional Animal Care and Use Committee (IACUC) of the University of Kentucky, Lexington KY. All methods were carried out according to relevant guidelines and regulations. Commercial male TALLYHO/JngJ (TH) mice (genetically homologous with the inbred SWR/J strain) were purchased from Jackson Laboratories (Bethesda, MD, USA) at ~ 6 weeks of age. A group of age-matched SWR/J mice [lean control; non-diabetic ND; normoglycemic NG] (Jackson Laboratories) were used as lean controls. Because the phenotype is less than 100% penetrant, only ~75% of male TH mice convert quickly to overt diabetes with elevated BG levels (BG>300 mg/dL) [TH-D mice; diabetic; convertors], while the remaining cohort (~25%) maintain lower BG values until the end of the study, corresponding to a pre-diabetes control condition [TH-Ctrl mice; pre-diabetic; non-convertors]. As observed in our mice colony, in adulthood, TH-Ctrl mice display moderate obesity and larger islets compared with the lean SWR mice. In contrast, mice with overt diabetes display significant β-cell loss and dwindling islets (Fig. 1A).

Mice from both TH groups (TH-D and TH-Ctrl mice) were randomized to receive standard rodent chow (Envigo #2018) or chow containing 100 ppm Canagliflozin (Sellecktchem, TX) for 10 weeks (TH-Ctrl; TH-D and, respectively TH-Ctrl/Cana; TH-D/Cana) (Fig.1B). The Cana dosing was calculated according to dose conversion guidelines (Nair & Jacob, 2016) so it corresponds to 20 mg/kg/day of drug intake (which would be comparable to the human canagliflozin therapeutic dose of 100 mg/day). In the end of the study, mice were euthanized by decapitation under deep isoflurane anaesthesia, fasting blood glucose (FBG) levels were determined on trunk blood and blood samples were collected immediately on ice and served for biochemical assay of fasting insulin (FI), C-peptide and glucagon; tissues were collected and snap-frozen in liquid nitrogen. Before euthanasia, mice were fasted 16 hours (over the night; O/N) to measure fasting blood glucose (FBG) and corresponding plasma insulin (FI) and glucagon levels. The fasting/refeeding experiment was performed on another cohort of mice. During the experiment, all mice were housed in the same animal room with a 14:10-hour light-dark cycle and free access to food and water. Mice were weighed and screened every other week for random non-fasting blood glucose levels in the whole blood from the lateral tail vein, using an ΑTRAK2 glucometer (Zoetis, MI).

**Immunohistochemistry**

Mouse pancreata were rinsed in cold sterile saline and fixed immediately after death in cold 4% paraformaldehyde solution (PFA4% in PBS) over the night at 4°C. Fixed tissues were paraffin-embedded and sections (5µm thickness) were mounted on Superfrost Plus Micro slides (VWR Int., PE). Sections were dewaxed, rehydrated, permeabilized, and blocked with 10% normal goat serum, preceded by microwave antigen retrieval in citrate buffer 10 mM (pH 6).  Sections were stained with mouse anti-insulin (Sigma-Aldrich; I2018; 1:400), guinea pig anti-insulin (Abcam; ab7842; 1:200), mouse anti-glucagon (Sigma-Aldrich; G2654; 1:200), rabbit anti-glucagon (Abcam; ab92517; 1:400-600), rat anti-somatostatin (Invitrogen ; MA5-16987; 1:100), rabbit anti- PDX1 (Abcam; ab47267; 1:100), rabbit anti-NKX6.1 (Novus Biologicals; NBP1-49672; 1:100), rabbit anti-PC1/3 (Millipore; AB10553; 1:100); rabbit anti-GLUT2 (MilliporeSigma; 07-1402; 1:100), rabbit anti-Ki67 (Biocare Medical; CRM325A; 1:200), rabbit anti-GLP1 (Abcam; ab22625; 1:200), rabbit anti-VIM (Abcam; AB92547; 1:100), rabbit anti-NGN3 (Novus; NBP2-27115SS; 1:100). Primary antibodies were detected with appropriate secondary antibodies conjugated with specific AlexaFluor fluorophores (ThermoFisher Scientific, MA). Nuclei were counterstained with Dapi (Millipore Sigma, MO). Stained slides were inspected with an inverted AxioObserver Z1 microscope (Carl Zeiss AG, Germany) equipped with the AxioCam506 Mono Camera and digitized with the ZEN2.3Pro software; ZEN3.4lite and ImageJ 1.52a were used for image analysis.

**Super-resolution microscopy**

Pancreas sections mounted using high precision No. 1.5 coverslips (Zeiss, Jena) and the hardening antifade mountant Prolong Gold (Thermo Fisher Scientific, Waltham, MA) were inspected with a structured illumination microscope SIM (Nikon N-Sim, Melville, NY).

**Histomorphometry and quantifications**

Islet histomorphometry was assessed in Hematoxylin & Eosin (H&E) stained sections (5 µm thickness), as previously described ^14^. Other quantifications were conducted on immunofluorescence images in pancreas-stained sections from min. n = 3 mice per group (randomly assigned). At least three sections per mouse pancreas (at least 100 μm apart) were analyzed with ZEN3.4 lite or ImageJ 1.52a software (Bethesda, MD, https://imagej.nih.gov/ij/).

Nair, A. B., & Jacob, S. (2016). A simple practice guide for dose conversion between animals and human. *Journal of basic and clinical pharmacy*, *7*(2), 27–31. https://doi.org/10.4103/0976-0105.177703
